# Supplementary material for: Nanostructured Electrospun Fibers with Self-Assembled Cyclo-L-Tryptophan-L-Tyrosine Dipeptide as Piezoelectric Materials and Optical Second Harmonic Generators
Source: Materials (Basel). 2023 Jul 14;16(14):4993. doi: 10.3390/ma16144993 (PMC10384039; doi:10.3390/ma16144993)
Supplement: Supplementary file 1 [file materials-16-04993-s001.zip › materials-2489260-supplementary.pdf]

materials

MDPI

Article

Nanostructured Electrospun Fibers with Self-Assembled Cyclo-L-Tryptophan-L-Tyrosine Dipeptide as Piezoelectric Materials and Optical Second Harmonic Generators

Daniela Santos [1], Rosa M. F. Baptista [1,*], Adelino Handa [1], Bernardo Almeida [1], Pedro V. Rodrigues [2], Cidália Castro [2], Ana Machado [2,*], Manuel J. L. F. Rodrigues [1], Michael Belsley [1] and Etelvina de Matos Gomes [1]

[1] Laboratory for materials and Emergent Technologies (LAPMET), Centre of Physics of Minho and Porto Universities (CF-UM-UP), University of Minho, Campus de Gualtar, 4710-057 Braga, Portugal
[2] Institute for Polymers and Composites, University of Minho, Campus de Azurém, 4800-058 Guimarães, Portugal
* Correspondence: rosa_batista@fisica.uminho.pt and avm@dep.uminho.pt

Supplementary Information

S1. Piezoelectric Measurements: Setup

Figure S1 shows the experimental setup for piezoelectric measurements. The red numbers, in Figure S1 a, represent the different components of the assembly: (1) is where the sample is placed, the forces are uniformly applied to the fiber mat by a membrane; (2) oscilloscope to measure the output voltage; (3) frequency generator and (4) preamplifier.

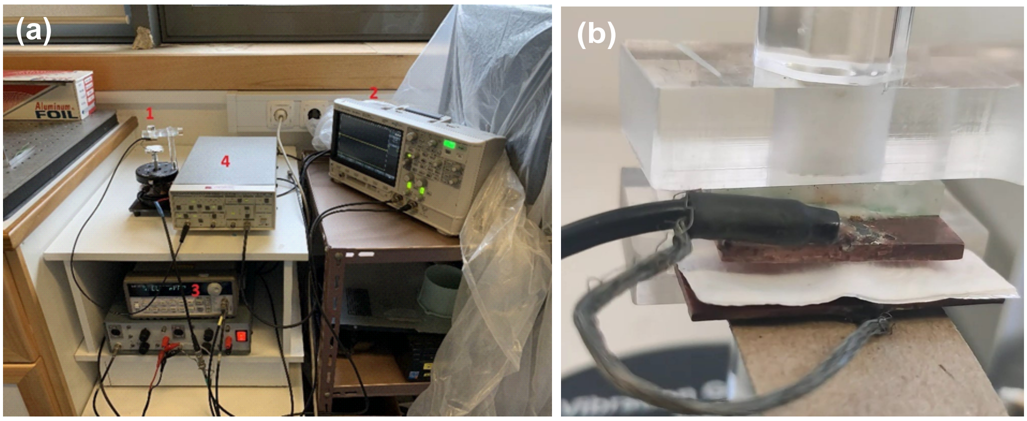

Figure S1. **(a)** Experimental setup for piezoelectric measurements. In **(b)** is shown the fiber mat (white) placed between the copper electrodes.

S2. Attenuated Total Reflectance Fourier Transform Infrared Spectroscopy (FTIR-ATR)

Fourier transformed infrared analysis of electrospun PCL, Cyclo(L-Trp-L-Tyr)@PCL fibers and Cyclo(L-Trp-L-Tyr) powder was recorded on a Jasco 4100 FTIR spectrometer in transmittance mode, in the range of 4000 to 600 cm^{-1}, averaging 32 scans and using a resolution of 8 cm^{-1}. FTIR data were treated with OriginPro 2018 SR1 software (OriginLab Corporation, Northampton, MA, USA).

As depicted in Figure S2, after the integration of the cyclo-dipeptide into the PCL fibers, a reduction in intensity is evident in the FTIR spectra of the dipeptide peaks. This phenomenon can be attributed to the intermolecular interaction occurring between the dipeptide and the polymeric matrix. Nevertheless, no noteworthy disparities in the vibrational frequencies of these peaks were detected, signifying that the structural integrity of the

cyclo-dipeptide remains intact subsequent to its self-assembly into nanotubes encapsulated within the PCL nanofibers.

The reduction in intensity of the FTIR peaks can be attributed to the transfer of vibrational energy from the cyclo-dipeptide to the polymeric matrix. This molecular interaction leads to a decrease in the energy absorption of the sample, consequently resulting in diminished intensity of the peaks observed in the FTIR spectra. An additional factor to consider is that electrospun nanofibers consist of a dipeptide:polymer ratio of 1:5, which may contribute to the diminished intensity observed in the peaks.

Certain peaks within the range of 1100 – 3400 cm^{-1} were successfully assigned. The stretching band of the amide group (C = O) in the peptide was observed at 1660 cm^{-1}, corresponding to a β-sheet arrangement of Cyclo(L-Trp-L-Tyr) molecules [28,43]. The stretching band of the N–H bond in Cyclo(L-Trp-L-Tyr) monomers appears as a narrow peak at 3363 cm^{-1} and as a broad and less intense band when self-assembled within PCL fibers (Cyclo(L-Trp-L-Tyr)@PCL), attributed to the N–H/O–H stretching vibrations, between cyclo-dipeptide molecules (L-Trp-L-Tyr). At approximately 1107 cm^{-1}, a peak indicative of the C = C stretching vibration is observable, while another peak can be observed at 1234 cm^{-1} corresponding to the C–N stretching vibration [27,28].

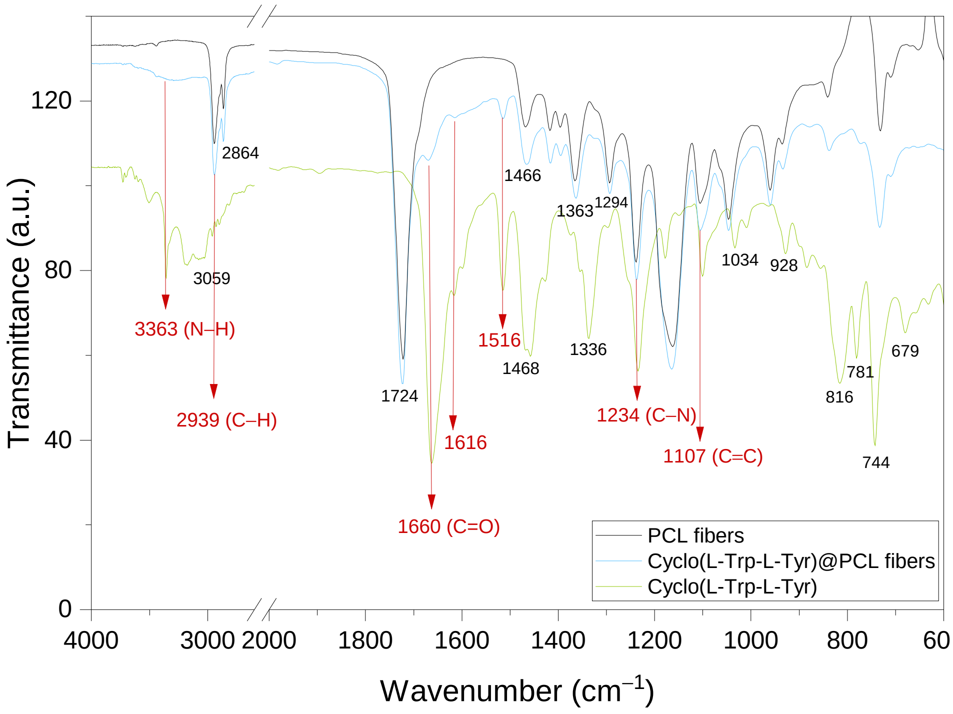

Figure S2. FTIR spectra of the Cyclo(L-Trp-L-Tyr) powder, the polymer (PCL) and Cyclo(L-Trp-L-Tyr)@PCL nanofibers.

S3. Thermogravimetric Analysis (TGA) and Differential Scanning Calorimetry (DSC)

Thermogravimetric analysis (TGA) of the fibers and powder was accomplished using a TGA Q500 (TA Instruments, New Castle, USA) under nitrogen atmosphere at 10 °C/min in a temperature range from 40 to 600 °C. The thermal behavior was analyzed using a DSC Netzsch 200 Maya (Netzsch, Selb, Germany). The tests were carried out under nitrogen atmosphere with a heating rate of 2 °C/min from 25 to 200 °C.

The TGA spectrum of the Cyclo(L-Trp-L-Tyr) dipeptide reveals a small initial mass loss of 3% occurring at 108 °C, corresponding to the loss of residual water. Above 393 °C, a considerable mass loss of approximately 76% is observed, related to the degradation of the dipeptide. The fusion followed by complete degradation of the PCL fibers, resulting in a mass loss of 97%, occurs at 405 °C, as reported previously [13,38,44]. The PCL fibers incorporated with the dipeptide exhibit complete degradation at 397 °C, with a mass loss

of 95%, indicating a slight decrease in thermal stability compared to the PCL fibers without the dipeptide, see Figure S3.

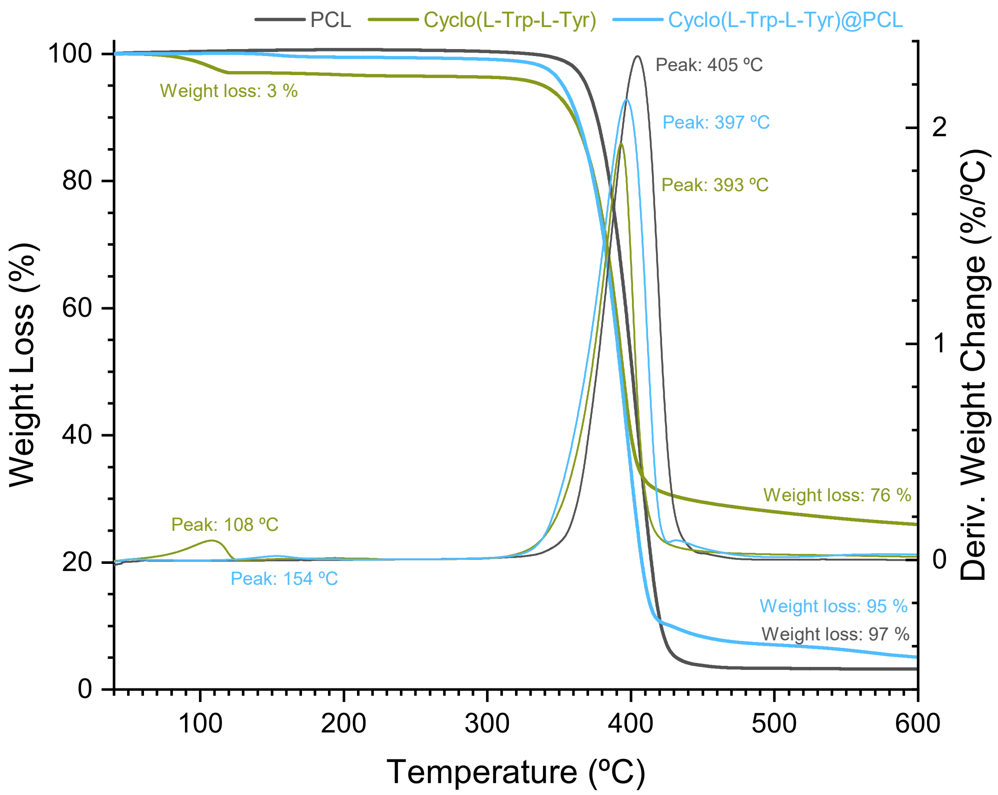

Figure S3. TGA spectra of Cyclo(L-Trp-L-Tyr) dipeptide, Cyclo(L-Trp-L-Tyr)@PCL and PCL fibers.

Figure S4 shows the DSC analysis, it was determined that the incorporation of the dipeptide had no significant effect on the glass transition temperature (T_g) of PCL, which occurs for both at around 60 °C, this value is corroborated in literature [44]. The dipeptide remains stable within the measured temperature range and does not exhibit any changes in physical state, such as solid-liquid phase transitions or alterations in molecular or crystalline arrangement.

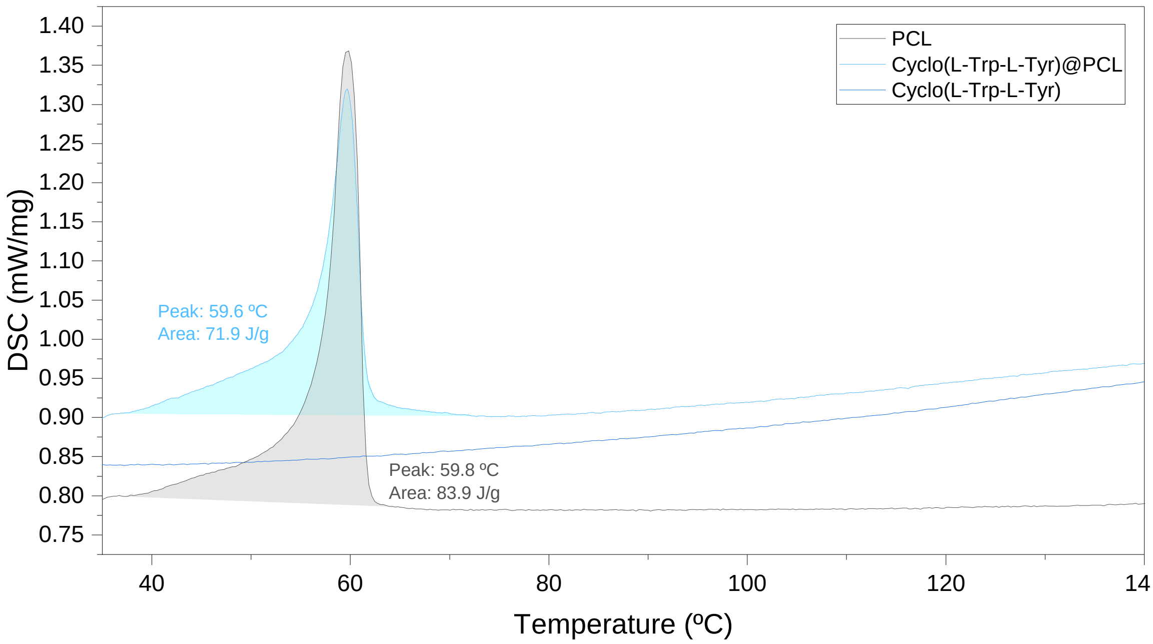

Figure S4. DSC spectra of Cyclo(L-Trp-L-Tyr) dipeptide, Cyclo-(L-Trp-L-Tyr)@PCL and PCL fibers.
